# Variability of unilateral and bilateral isometric muscle strength of lower extremities extensors in young females and males

**DOI:** 10.1186/s13102-023-00795-0

**Published:** 2024-01-02

**Authors:** Jaroslaw Kabacinski, Michal Murawa, Tadeusz Wojtkowiak, Krzysztof Mackala, Lechoslaw B. Dworak

**Affiliations:** 1 Department of Biomechanics, Poznan University of Physical Education, Poznań, Poland; 2 Department of Dance and Gymnastics, Poznan University of Physical Education, Poznań, Poland; 3 Department of Track and Field, Wroclaw University of Health and Sport Sciences, Wrocław, Poland; 4 Faculty of Medicine and Health Sciences, Calisia University - Kalisz, Kalisz, Poland

**Keywords:** Lower extremity extensors, Isometric muscle strength, Gender, Closed kinetic chain, Knee extension angle, Strain gauge dynamometry

## Abstract

**Background:**

The muscle strength of the lower extremity extensors can be evaluated in the closed kinetic chain (CKC) during unilateral or bilateral conditions. Factors such as the mass and length of the muscle, joint angle, type of contraction, and gender influence the magnitude of the muscle strength. The aim of this study was to compare the isometric strength of lower extremity extensors between the different knee extension angles (KEs) as well as between bilateral and unilateral conditions.

**Methods:**

Nineteen female students (age: 20.2 ± 0.6 years) and nineteen male students (age: 20.3 ± 0.7 years) participated in the study. The muscle strength was evaluated in CKC using the strain gauge dynamometer. The analysis included values of the maximum muscle strength normalized to body mass (MS/BM) for the six KEs of 80°, 70°, 60°, 50°, 40° and 30°.

**Results:**

A significant main effect in the MS/BM values for the angle factor (*p* < 0.001) and condition factor (*p* < 0.001) was found. Moreover, there was a non-significant interaction effect between the angle factor and gender factor (*p* = 0.476) as well as between the condition factor and gender factor (*p* = 0.770). Comparisons showed significant differences in the MS/BM values between the six KEs (*p* < 0.001). Furthermore, significantly lower MS/BM values for bilateral conditions than unilateral conditions at the 30° KE were observed (*p* < 0.001).

**Conclusion:**

The decrease in KE by 10° significantly increased the muscle strength of the lower extremity extensors. Gender did not affect the change in MS/BM values with the change in KE and conditions. Findings also revealed significant bilateral deficit, i.e., significantly a lower summed muscle strength during bilateral conditions than unilateral conditions. The study emphasized the importance of selecting the 30° KE as the optimal angle to assess the maximum strength developed in CKC.

## Background

Muscle strength depends on muscle length, i.e., the value of the resultant of the active and passive strength for the isometric contraction increases with muscle stretching to maximum when muscle is partially stretched [1]. The change in muscle strength with the change in joint angle indirectly indicates the strength-length relationship [2]. In the case of the knee joint, the variation in isometric quadriceps torque in a range of motion was examined, indicating a knee angle in the range 60°-80° for developing maximum strength [3–6]. Other studies reported increase in the values of the isometric strength of LE extensors with decrease in the knee extension angle (KE) from 100° to 40° and hip extension angle (HE) from 80° to 50° in both gender subjects [7], and the KE from 75° to 30° and HE from 130° to 90° in male students [8].

Muscle strength can be examined in a closed kinetic chain (CKC) during isometric contraction. The test in CKC provides an assessment of the maximum strength of lower extremity (LE) extensors developed at the set knee joint angle in bilateral or unilateral conditions [9–12]. The importance of testing this strength is emphasized by the occurrence of LEs muscle loads in CKC, e.g., during various types of squats, cycling or rowing.

For the tests in CKC, it is also possible to determine the bilateral deficit (BD) index, defined as the ratio of strength produced by both limbs simultaneously to the summed strength produced by each limb independently [9, 13–15]. The result of this index below 100% indicates BD, thus a lower bilateral strength than a summed unilateral strength. One of the presumable causes of BD includes the mechanism of the reduction in motor unit excitability during bilateral conditions [13, 16, 17]. In addition, the magnitude of BD index is related to movement patterns [18], gender [19], unilateral or bilateral exercises [9, 20] as well as the level of competition in the selected sports [21].

Some authors examined the effect of the different KEs on the isometric strength of the knee extensors and flexors in females and males [3, 4] as well as the effect of gender on the change in thigh muscle strength from the KE [3]. However, these studies evaluated differences in the quadriceps and hamstrings peak torque tested in the open kinetic chain (OKC). Analysis of the available studies indicated insufficient data on the variation of the unilateral and bilateral strength developed in CKC by the LEs extensors among subjects of both genders. Therefore, the aim of this study was to compare the isometric muscle strength of LE extensors between the different KEs as well as between bilateral and unilateral conditions in female and male students.

## Methods

### Participants

Nineteen untrained healthy female students (age: 20.2 ± 0.6 years, body mass: 56.8 ± 5.4 kg and body height: 1.68 ± 0.05 m) and nineteen untrained healthy male students (age: 20.3 ± 0.7 years, body mass: 74.2 ± 3.9 kg and body height: 1.81 ± 0.03 m) with Poznan University of Physical Education participated in the study (mean ± standard deviation). All participants fulfilled the following inclusion criteria: (1) age between 18 and 24 years, (2) recreational physical activity, (3) lack of potential medical problems, (4) lack of history of the ankle, knee, hip or back injuries in the one year before the testing. Participants undertook moderate-intensity aerobic physical activity including mainly running, cycling, roller skating and swimming for at least 300 min per week. According to the guidelines, participants did not engage in high-intensity physical activity 48 h prior to testing. Each student was acquainted with the experimental procedures and provided written informed consent to participate in the research. The study received approval from the Bioethical Committee at the Poznan University of Medical Sciences (number 546/16) in accordance with the Declaration of Helsinki.

### Experimental procedures

**Fig. 1:**
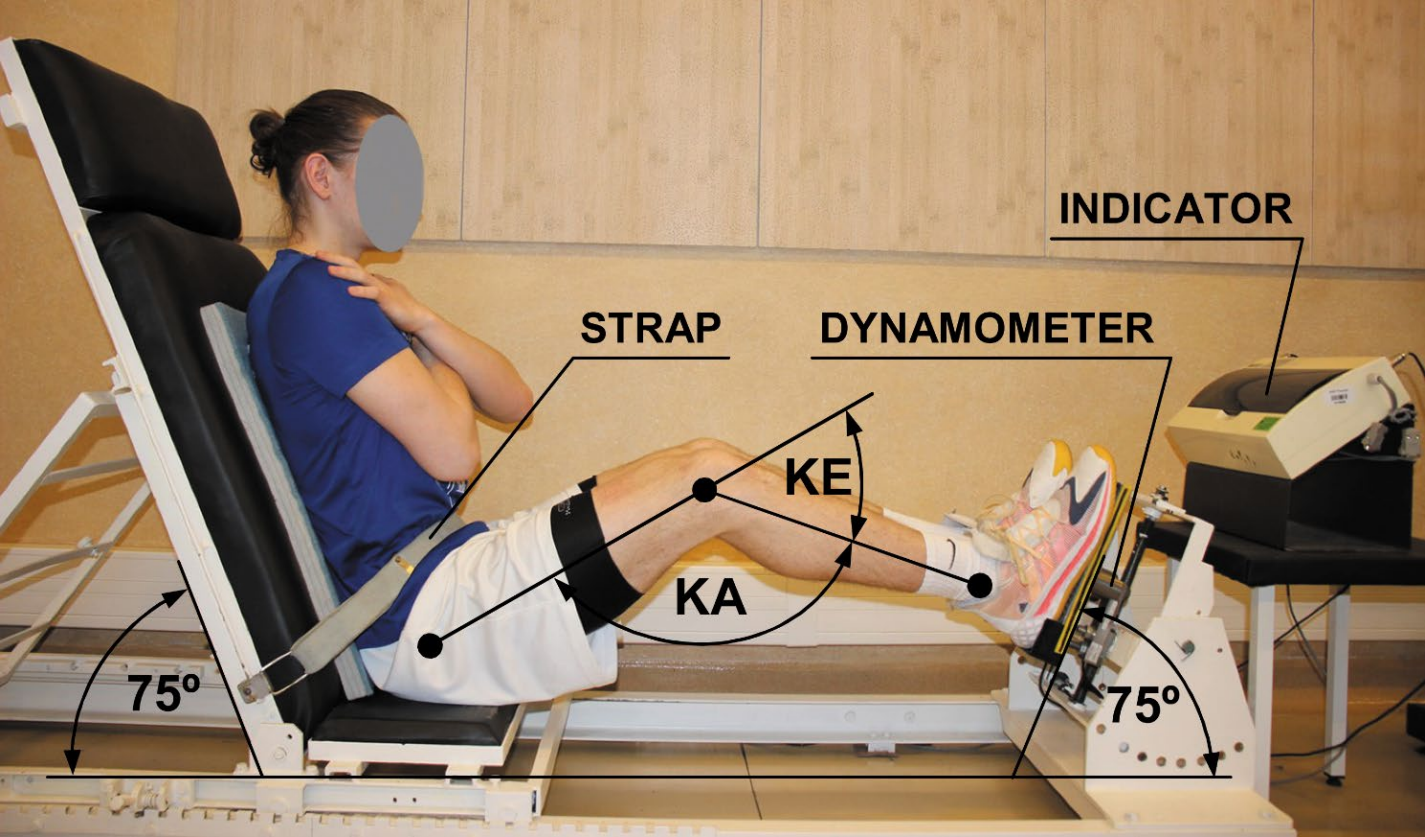
Test of the lower extremity extensors strength developed in the closed kinetic chain (KA—knee angle, KE—knee extension angle). Written consent was provided by participant

The isometric strength of LE extensors developed in CKC was examined during the 4-day period, always from 11am to 3pm. The tests were conducted using the Strength Measuring Station-1 (SMS-1) device with a sliding chair mechanism. The SMS-1 was equipped with the strain gauge dynamometer Scaime SB30X (measurement error ± 0.017%) and the indicator PUE 1 (RADWAG Company, Radom, Poland) (Fig. 1).

Before the test, each subject performed five minutes of a total body warm-up by cycling on the stationary bike (Monark Ergomedic 874E) followed by five minutes of muscle stretching. The student sat in the position with hands held across the chest during testing. The pelvis was restrained by a stabilizing strap. The backrest and foot pedals were inclined at the 75 ± 1° angle. The tests were performed at a set knee angle of 100°, 110°, 120°, 130°, 140°, 150° by a standard goniometer (measurement error ± 1°). The change in angle was provided by sliding chair of the SMS-1 device. The knee angle was always determined regarding the hip rotation axis, knee rotation axis and ankle by the same experienced investigator. The measured knee angle corresponded to the anatomical KE of 80°, 70°, 60°, 50°, 40° and 30°, respectively (0° = full knee extension). During the test, the subject pressed the right foot on the pedal, developing the maximum isometric strength (maximum voluntary contraction) of LE extensors for 3 seconds in 2 repetitions for each KE. The opposite LE rested on the ground. Then, for the KE of 30°, the subject developed the maximum isometric strength for 3 seconds during extension of both LEs simultaneously (bilateral conditions) in 2 repetitions and of the left LE (2 repetitions), and of the right LE (2 repetitions) independently (unilateral conditions). Measurements were preceded by one trial repetition with sub-maximal effort for each of the six KEs. The rest period between the repetitions was approximately one minute. Participants were verbally encouraged to develop maximum strength with the command „press with maximum strength”. The analysis included results of the maximum strength normalized to body mass (MS/BM) of LE extensors obtained during the best repetition for: (1) each KE (values of MS/BM for the right LE), (2) bilateral and unilateral conditions (summed values of MS/BM for both LEs, respectively) at the KE of 30°. Percentage differences for the mean MS/BM values between the KE were calculated using the formula:

1 $$ $$\frac{{{X_1} - {X_2}}}{{{X_1}}} \cdot 100\% $$ $$

where, X_1_ > X_2_ and X_1_, X_2_ – MS/BM values. In turn, BD of LEs extensors strength was calculated using the formula:

2 $$ $$100\%- \frac{{{{({X_1} + {X_2})}_{bilateral}}}}{{{{({X_1} + {X_2})}_{unilateral}}}} \cdot 100\% $$ $$

where, X_1_ – MS/BM value for the right LE, X_2_ – MS/BM value for the left LE.

### Statistical analysis

Statistical analysis was conducted using the IBM SPSS Statistics software for Windows, version 28.0 (Armonk, NY, USA: IBM Corp). The distribution of the data was verified via the Shapiro-Wilk test. The independent samples *t*-test for the characteristics of students and the mixed-factorial ANOVA with two factors (angle [80°, 70°, 60°, 50°, 40° or 30°] × gender [female or male]) and (condition [bilateral or unilateral] × gender [female or male]) for the MS/BM values were performed. Sphericity was determined using the Mauchly test. The Greenhouse-Geisser adjustment was made when sphericity was violated. The Bonferroni correction was used to compare within-subjects factors. Statistical power and effect sizes were calculated. According to the Cohen guidelines for the ANOVA effect size, values of the partial eta-squared (η^2^) were small for 0.01, medium for 0.06 and large for 0.14 [22]. Significance level alpha was defined at *p* < 0.5.

## Results

Significantly higher values of body height (by 7.2%; *p* < 0.001), body mass (by 23.5%; *p* < 0.001) in males than females were found. However, the age difference between females and males was not significant (0.5%; *p* = 0.642). Statistical power was 1.0 for the MS/BM values in both females and males (with a sample size of 19 subjects).

Table 1 presents the means ± standard deviations of the LE extensors strength.

**Table 1:** Means ± standard deviations of the lower extremity extensors strength for the six knee flexion angles in females and males

Angle [°]	Strength [N∙kg^− 1^]	
	Females	Males
80	13.7 ± 2.2	14.9 ± 2.3
70	15.3 ± 2.4	17.2 ± 3.1
60	18.2 ± 2.9	20.1 ± 3.8
50	21.4 ± 3.6	24.2 ± 3.9
40	27.0 ± 5.1	29.8 ± 4.6
30	30.9 ± 6.4	33.5 ± 5.3

The analysis revealed a significant main effect in the MS/BM values for the angle factor (F_2,64_ = 330.8; η^2^ = 0.902; *p* < 0.001) and a non-significant main effect for the gender factor (F_1,36_ = 4.1; η^2^ = 0.102; *p* = 0.061). Moreover, a non-significant interaction effect (F_2,64_ = 0.718; η^2^ = 0.020; *p* = 0.476) between the angle factor and gender factor was found. The results of the Greenhouse-Geisser adjustment (0.355) indicated that the condition of sphericity was fulfilled.

The *p*-values of the post-hoc test for the comparisons of LE extensors strength between the KEs are presented in Table 2.

**Table 2:** The means ± standard deviations of the differences and *p*-values for comparisons of the lower extremity extensors strength between the knee flexion angles

Angle [°]	Females			Males	
	Diff [%]	*p*		Diff [%]	*p*
70 vs. 80	10.2 ± 1.9*	< 0.001		12.7 ± 3.8*	< 0.001
60 vs. 70	15.8 ± 2.5*	< 0.001		14.3 ± 3.7*	< 0.001
50 vs. 60	14.9 ± 3.6*	< 0.001		17.1 ± 3.9*	< 0.001
40 vs. 50	20.1 ± 4.1*	< 0.001		19.0 ± 3.2*	< 0.001
30 vs. 40	12.3 ± 4.1*	< 0.001		10.9 ± 3.3*	< 0.001

Diff–difference in the lower extremity extensors strength between the angles, *–significant difference (*p* < 0.05)

Comparisons between the angles showed significantly higher strength values for: (1) 70° than 80°, (2) 60° than 70°, (3) 50° than 60°, (4) 40° than 50° and (5) 30° than 40° both in females and males (*p* < 0.001). The mean differences between females and males for the values in Table 2 were non-significant (1.1–2.5%; *p* > 0.05).

Table 3 shows the *p*-values of the post-hoc test for the comparisons of LE extensors strength between the females and males.

**Table 3:** The means ± standard deviations of the difference and *p*-values for comparisons of the lower extremity extensors strength between the females and males

Angle [°]	Females vs. males	
	Diff [%]	*p*
80	7.9 ± 5.3	0.109
70	10.6 ± 5.1*	0.046
60	9.1 ± 4.4	0.091
50	11.4 ± 4.2*	0.031
40	10.0 ± 6.6	0.077
30	8.4 ± 7.8	0.172

Diff—difference in the lower extremity extensors strength between the females and males, *—significant difference (*p* < 0.05)

The analysis demonstrated significantly greater strength values in males than females for angles: (1) 70° and (2) 50° (*p* < 0.05).

**Fig. 2:**
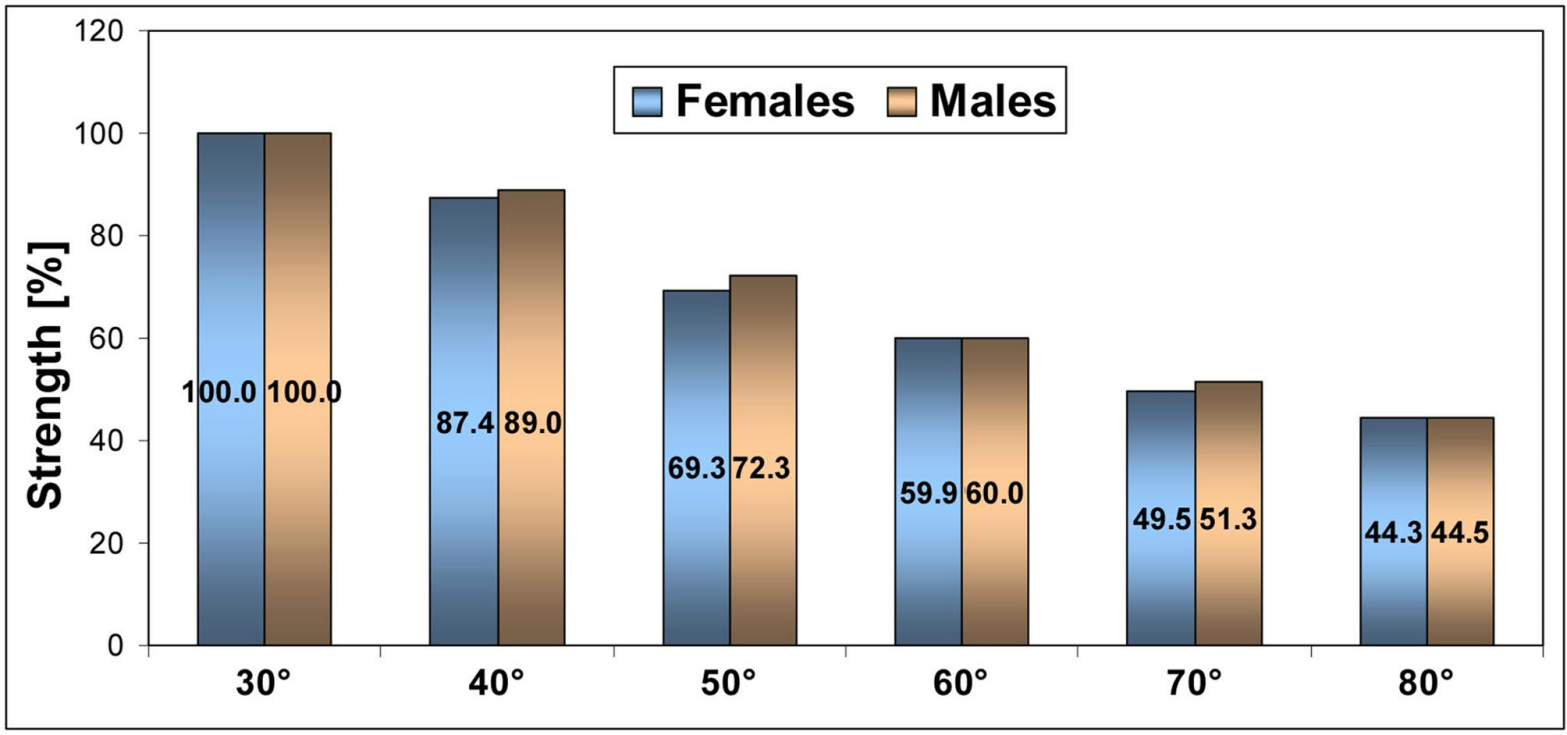
Percentage values of the lower extremity extensors strength for the six knee extension angles in females and males

Assuming 100% of MS/BM at the KE of 30°, were significantly lower values (*p* < 0.05) by: (1) 12.6% (40°), 30.7% (50°), 40.1% (60°), 50.5% (70°) and 55.7% (80°) in females as well as (2) 11.0% (40°), 27.7% (50°), 40.0% (60°), 48.7% (70°) and 55.5% (80°) in males (Fig. 2).

**Fig. 3:**
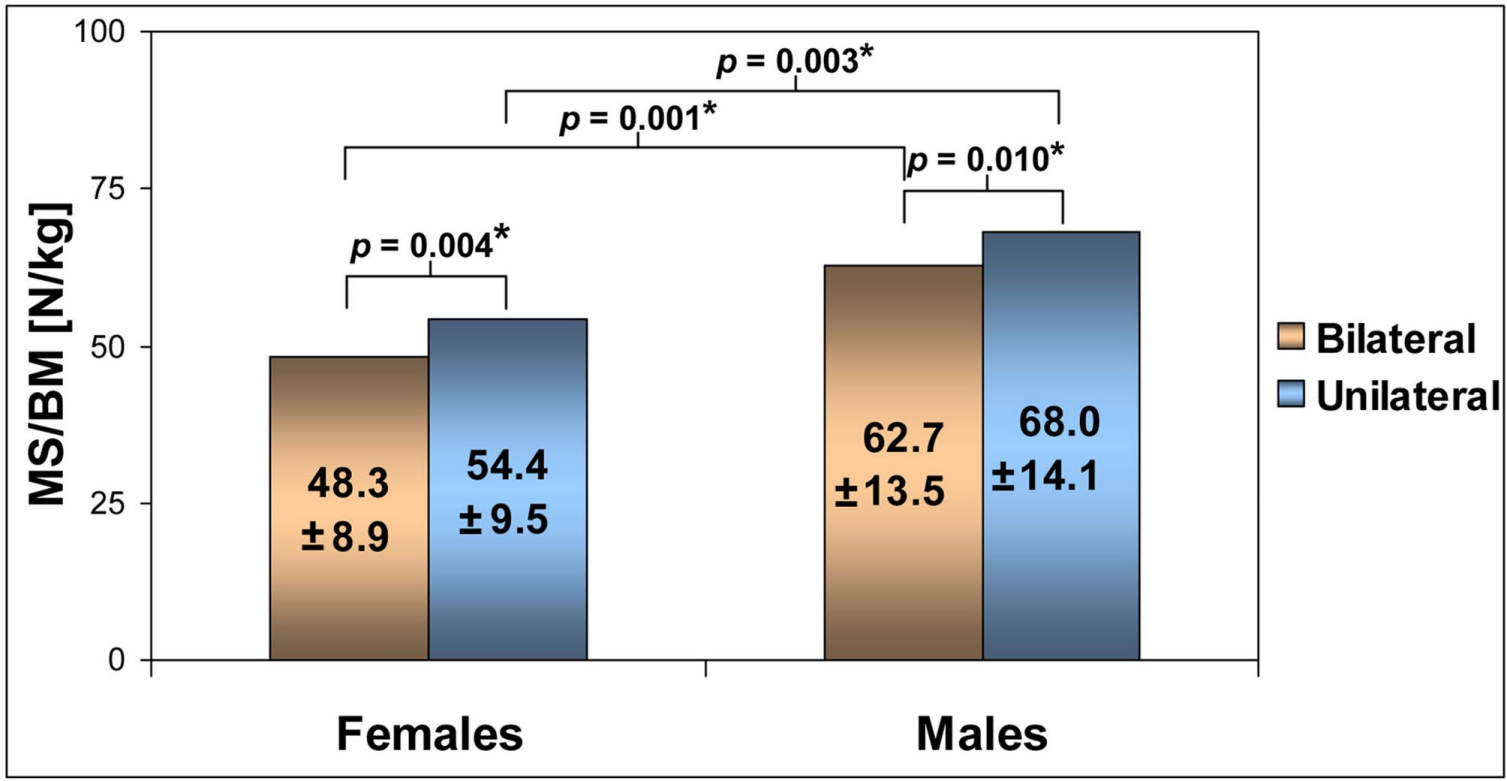
Means ± standard deviations of the lower extremity extensors strength at the knee extension angle of 30° for bilateral and unilateral conditions and p*-*values for the comparisons between conditions and between females and males (MS/BM—maximum muscle strength normalized to body mass, *—significant difference)

There was a significant main effect in the MS/BM values for the condition factor (F_1,30_ = 17.4; η^2^ = 0.368; *p* < 0.001) and for the gender factor (F_1,30_ = 12.9; η^2^ = 0.301; *p* = 0.001). Moreover, a non-significant interaction effect (F_1,30_ = 0.718; η^2^ = 0.003; *p* = 0.770) between the condition factor and gender factor was found. The results of the Mauchly test (W = 1,0; *p* > 0.05) indicated that the condition of sphericity was fulfilled.

Pairwise comparisons revealed significantly lower MS/BM values (*p* < 0.001) for bilateral conditions than unilateral conditions by 11.2% in females and by 7.8% in males. Significantly greater MS/BM values in males than females were found by 23.0% for bilateral conditions (*p* = 0.001) and by 20.0% for unilateral conditions (*p* = 0.003) (Fig. 3). Moreover, the mean difference between females and males for the BD values was non-significant (3.4%; *p* > 0.05).

## Discussion

The present study compared the isometric strength of LE extensors developed in CKC between the KEs in female and male students. Findings revealed significant differences in the MS/BM values between the six KEs (80°, 70°, 60°, 50°, 40° and 30°). Hence, the decrease in KE by 10° significantly increased the strength values from 13.7 N kg^− 1^ (females) and 14.9 N kg^− 1^ (males) for 80° to 30.9 N kg^− 1^ (females) and 33.5 N kg^− 1^ (males) for 30°. Although, it was not expected that a change in angle by 10° would cause a significant improvement in strength. Therefore, these results may play an important role in selecting the appropriate knee angle when evaluating the maximum strength of the LE extensors.

Previous studies also evaluated variation in the LE extensors strength developed in CKC [7, 8]. For example, Wojtkowiak et al. [7] reported the influence of LEs joint angles on the isometric strength of LE extensors and the greatest values of the isometric strength of LE extensors at the KE in the range 40°-50° and HE in the range 70°-80° in women and men. Moreover, Urbanik et al. [8] found an increase in the ground reaction force with the decrease in the KE from 75°to 30° and HE from 130° to 90° for both LEs of students. However, it was only this study that compared the isometric strength of LE between the KEs and determined the influence of gender on the strength differences between these angles.

In addition to the MS/BM comparisons between the angles, the influence of gender on the change in strength with increasing angle was also determined. The analysis showed a non-significant main effect, i.e., non-significant differences between women and men as well as significantly higher MS/BM values in males than females only at 50° and 70° angles. Importantly, a non-significant interaction between the angle and gender factors was demonstrated; the gender factor did not influence the variation of muscle strength from the KE. Thus, for the isometric test of LE extensors strength in CKC, change in MS/BM values from the KE does not depend on gender. In comparison, Kong and Burns [3] also examined the effect of gender on the variation in quadriceps peak torque with the change of the KE. In contrast to these findings, Kong and Burns [3] demonstrated significantly higher values of the overall quadriceps torque in males than females and a significant angle-gender interaction. However, these researchers evaluated muscle strength in OKC.

Several studies compared the isometric quadriceps strength developed in OKC between the different KE for both females and males [3, 4]. The highest values of the knee extensors peak torque were found at 80° [3] and at 60° [4]. In turn, the present study revealed the highest MS/BM values of the LEs extensors at the KE of 30°. Thus, maximum muscle strength of the knee extensors and hip extensors in CKC is developed at the smaller KE than for the quadriceps peak torque measured in OKC, i.e., when muscles are less stretched. This difference results from a different sitting position of the subject (different position of the thigh and lower leg) during the test in CKC and the production of muscle strength by both the knee and hip extensors.

This study also compared the values of bilateral and unilateral muscle strength developed by the LEs extensors at the optimal KEs of 30° between females and males. Findings showed a significant between-subject main effect and significantly higher MS/BM values in men than women, as well as a non-significant interaction effect between the condition and gender factors. Hence, the increase in LEs extensors strength for unilateral conditions compared to bilateral conditions does not depend on gender.

Considering the comparisons between the bilateral conditions and unilateral conditions, it was observed significantly a lower summed muscle strength developed by extensors of both LEs simultaneously than of both LEs independently, i.e., BD of approximately 11% in females and approximately 8% in males. Similarly, other studies demonstrated significant BD of knee extensors strength in recreationally active young women [20] and healthy young men [23]. In contrast, Bulzing et al. [24] reported BD values close to zero in volunteers; however, this was most likely caused by intrinsic random error of measurement.

For the isometric contraction of LEs muscles, some authors suggested possible causes of this deficit, such as the neural inhibition mechanism leading to the decrease in muscle strength produced bilaterally [23, 25, 26], difference in antagonist muscle coactivation between the bilateral contraction and unilateral contraction [27], and the reduction in motor neurons’ excitability during the bilateral conditions [13, 16, 17]. In addition, other authors indicate the body adjustments and mechanical configuration of the dynamometer as factors of BD [28]. The magnitude of BD can be increased by using a strength training program incorporating mainly unilateral knee extension exercises [20]. For example, Botton et al. [20] showed a greater increase in unilateral isometric strength in females after unilateral training than in females after bilateral training, thus higher BD for the unilateral group compared to the bilateral group.

Assessment of the maximum strength of the LE extensors in CKC is an important part of strength capabilities control in active population athletes. Based on these data, the highest strength was developed by the subjects at the KE of 30°. Moreover, the MS/BM value was significantly higher compared to results of this variable for the lower angles. Thus, measurements in CKC should first include setting of the KE at 30° (e.g., using a goniometer) as the optimal angle for assessing the maximum isometric strength of the LEs extensors.

This study has limitations. First, due to the safety of the subjects, measurements were not performed for KEs lower than 30°. Developing maximum strength at angles close to 0° can lead to hyperextension in the knee joint and increase the injury risk of the joint structures. Second, it is not possible to extrapolate these findings to other muscle groups tested in OKC because only the LEs extensors strength in CKC was examined.

## Conclusions

Findings showed significant differences in the MS/BM values between the KEs as well as the highest MS/BM values at the KE of 30° for both females and males. Importantly, decreasing the KE by only 10° significantly increased the LE extensors strength. In addition, the gender factor did not significantly affect the increase in muscle strength with the decrease in KE. Comparisons between the bilateral and unilateral conditions at 30° KE showed significantly a lower summed muscle strength developed bilaterally than unilaterally in men and women, i.e., significant bilateral strength deficit. Furthermore, it was observed that the increase in LEs extensors strength for the unilateral conditions compared to the bilateral conditions does not depend on gender. This study emphasized the importance of the 30° KE setting as the optimal angle to assess maximum muscle strength developed by LE extensors in CKC in both females and males.

## Data Availability

Data supporting reported results can be found at Department of Biomechanics, Poznan University of Physical Education, Królowej Jadwigi 27/39, 61–871 Poznań, Poland, info: biomechanics.awf.poznan@gmail.com.

## Abbreviations

BD: Bilateral deficit
CKC: Closed kinetic chain
HE: Hip extension angle
KA: Knee angle
KE: Knee extension angle
LE: Lower extremity
MS/BM: Maximum muscle strength normalized to body mass
OKC: Open kinetic chain
SMS-1: Strength Measuring Station-1
